# Evolution of the α‐Glucosidase Gene Family in Apoidea

**DOI:** 10.1002/ece3.74342

**Published:** 2026-09-13

**Authors:** Jiaqi Li, Wenmi Wang, Yuanyuan Du, Junxia Jia, Zhaoming Wei

**Affiliations:** ^1^ College of Life Science Shaanxi Normal University Xi'an China

**Keywords:** α‐glucosidase, Apoidea, functional differentiation, gene family, positive selection

## Abstract

With more than 20,000 recognized species, the Apoidea is one of the most significant groups of pollinating insects, and their living environment is complex and diverse. The composition of nectar‐producing plants for Apoidea varies across different geographical regions, prompting this group to evolve a variety of adaptive traits at the morphological and physiological levels to enhance their survival. The adaptive evolution of digestive enzymes is crucial for efficient nutrient absorption in Apoidea; however, the molecular basis underlying the diversification of bee digestive enzymes remains unclear. This study analyzed 63 α‐glucosidase sequences from the GH13 family, representing the three subfamilies Hbg1, Hbg2, and Hbg3, across six families within Apoidea. Phylogenetic analysis indicates that the α‐glucosidase gene family underwent two divergence events during the early evolution of the Apoidea superfamily, giving rise to the GH13 family members Hbg1, Hbg2, and Hbg3; compared to the ancestral lineage, these three members exhibit significant divergence in both expression patterns and enzymatic functions. The GH13 genes in honeybees exhibit substantial structural diversity. Furthermore, clear functional divergence was observed among the subfamilies, accompanied by signatures of positive selection in specific lineages and codon sites. Twenty‐eight sites near the ligand‐binding region of α‐glucosidase are subject to positive selection and functional divergence, which may be related to the ligand‐binding and hydrolytic properties of α‐glucosidase. Overall, the α‐glucosidase gene family has undergone adaptive evolution driven by positive selection in Apoidea, leading to the expansion and diversification of this gene family.

## Graphical abstract

This study traces the origin of the α‐glucosidase gene family in bees back to approximately 140 million years ago, revealing that its evolutionary history closely aligns with the rise of angiosperms and coevolution. Furthermore, by identifying the core members Hbg1–3 and 28 key functional sites driven by positive selection, the study systematically elucidates how this gene family has adapted through evolutionary processes to meet the demands of complex foraging behaviors and social evolution.

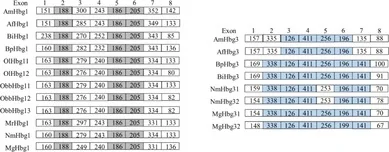

## 1 Introduction

The superfamily Apoidea belongs to the order Hymenoptera, suborder Apocrita, and infraorder Aculeata. More than 20,000 species of Apoidea have been recorded; the bee clade comprises seven extant families, whereas Apoidea sensu lato also includes apoid wasp lineages. In this study, we focused on six sampled bee families, namely Apidae, Andrenidae, Colletidae, Halictidae, Melittidae, and Megachilidae (Danforth et al. 2013; Sann et al. 2018). As important pollinators in terrestrial ecosystems, Apoidea play a crucial role in maintaining ecological balance and the diversity of angiosperms. The ecosystem services provided by Apoidea, particularly crop pollination, are of substantial importance to agricultural production, food and nutritional security, human health, and regional economies (Khalifa et al. 2021). Hymenopteran insects first appeared during the Triassic Period. Early symphytan hymenopterans laid their eggs within plant tissues, whereas the evolution of a constricted waist and a specialized ovipositor in Apocrita facilitated more diverse reproductive and ecological strategies. During the Cretaceous Period, Apocrita underwent extensive diversification, accompanied by pronounced abdominal constriction and functional differentiation of the body. Through long‐term selection for host plants, the pollen‐feeding superfamily Apoidea diverged from the burrowing bees of the suborder Apocrita, gradually evolving masticatory‐sucking mouthparts—unique among insects—that allow them to both chew solid pollen and suck liquid nectar, as well as specialized pollen‐collection structures such as pollen baskets on the hind legs and a pollen brush on the ventral surface of the abdomen. These morphological innovations enabled the superfamily Apoidea to efficiently utilize the pollen and nectar resources of angiosperms. Their origin (140–110 MYA) closely coincides with the emergence of early angiosperm pollen (139–131 MYA) and their subsequent rapid diversification, providing key evidence for studying the coevolution of insects and plants (Genise et al. 2020). Over the course of their long evolutionary history, Apoidea have developed multi‐level adaptive mechanisms at the morphological, physiological, and molecular levels: morphologically, they have evolved chewing‐sucking mouthparts, pollen baskets, and chemical receptors to enhance resource acquisition efficiency (Krenn 2019); physiologically, they respond to environmental threats through heat shock proteins and antioxidant enzyme systems (Yang et al. 2024); at the molecular level, the expansion of gene families (such as α‐glucosidases and odor‐binding proteins) and positive selection have driven dietary specialization (Li et al. 2017). These mechanisms include social behavior (Wcislo and Cane 1996), foraging habits (Neff and Simpson 1990; Struck 1994), pollen‐collecting habits (Cane and Buchmann 1989; Neff and Simpson 1988; Thorp 1979), morphological traits (Polidori et al. 2020), and molecular‐level factors (Forêt and Maleszka 2006; Torson et al. 2017; Xu et al. 2017). In this study, representative species from six families of *Apoidea* were selected, primarily to encompass the major phylogenetic lineages and to compare the evolutionary characteristics of the α‐glucosidase gene family under different life‐history and feeding strategies. The selected taxa included social and generally broadly feeding members of *Apidae*, as well as other members of *Apoidea* exhibiting solitary lifestyles, transitions toward sociality, and varying degrees of pollen specialization.

Carbohydrates are the primary energy source for insects. Research into their metabolic mechanisms not only provides a foundation for understanding insect digestive physiology but also offers a theoretical basis for developing insect management strategies. Through long‐term coevolution, plants have developed multiple defense mechanisms, including secondary metabolites, physical barriers, and nutritional deficiencies. Meanwhile, insects have effectively broken down various sugars in plant tissues by expanding the substrate specificity and optimizing the activity of carbohydrate‐digesting enzymes (such as α‐glucosidase), thereby successfully overcoming the nutritional barriers posed by their hosts (Li et al. 2017). α‐Glucosidase belongs to the glycoside hydrolase (GH) superfamily and is an evolutionarily conserved hydrolase widely found in various groups, including bacteria, fungi, arthropods, and vertebrates. In insects, this enzyme is primarily distributed in digestive tissues such as the midgut, where it catalyzes the hydrolysis of starch and oligosaccharides to provide energy (Seddigh and Darabi 2015). α‐Glucosidase acts on α‐1,4‐glycosidic bonds, catalyzing the hydrolysis of oligosaccharides to produce D‐glucose and plays a crucial role in the glucose metabolism of organisms (Chiba 1997).

Based on the amino acid sequence similarity of their catalytic domains, α‐glucosidases are classified into five glycoside hydrolase (GH) families: GH4, GH13, GH31, GH97, and GH122; among these (Gabriško 2013). Insect α‐glucosidases primarily belong to the GH13 family, which, together with the GH70 and GH77 families, forms the GH‐H superfamily, also known as the α‐amylase family (Janeček and Gabriško 2016). Research on insect α‐glucosidases has been documented in several model species: seven genes were identified in *Drosophila melanogaster* , and their tissue‐specific expression patterns were revealed (Gabriško and Janeček 2011); a novel α‐glucosidase capable of specifically hydrolyzing sucrose was discovered in *Ephestia kuehniella*; and in *Bombyx mori* , it was demonstrated that this enzyme system has evolved to adapt to the strongly alkaline environment of the midgut (Pytelková et al. 2009). In summary, α‐glucosidases exhibit complex functional diversification across different insect groups through gene duplication, specialized expression patterns, and alterations in catalytic properties.

At present, studies of α‐glucosidases in Apoidea have focused primarily on *Apis mellifera* . The major α‐glucosidase‐encoding genes identified in honey bees include Hbg1, Hbg2, and Hbg3, which exhibit marked differences in tissue expression patterns and enzymatic properties. Hbg1 and Hbg2 are predominantly expressed in the midgut, whereas Hbg3 shows relatively low expression in the midgut and is primarily expressed in the hypopharyngeal glands. In addition, Hbg2 expression can also be detected in the hemolymph, suggesting that it may be involved in physiological functions other than digestion. Hbg3 may be secreted from the hypopharyngeal glands into nectar, where it hydrolyzes sucrose and thereby contributes to carbohydrate digestion and honey formation (Ueno et al. 2015). In addition to differences in tissue expression patterns, Hbg1, Hbg2, and Hbg3 also differ markedly in substrate specificity and optimal reaction conditions. Hbg1 can hydrolyze sucrose and maltose, with an optimal pH of 5.0 (Kimura et al. 1990). In addition to hydrolyzing sucrose and maltose, Hbg2 exhibits α‐amylase activity and can hydrolyze soluble starch to produce maltose and maltooligosaccharides; it can also hydrolyze α‐1,6‐glycosidic linkages in substrates such as isomaltose, with an optimal pH of 5.0 (Takewaki et al. 1993). Hbg3 can hydrolyze sucrose and maltose, with an optimal pH of 5.5 (Nishimoto et al. 2001). These studies indicate that Hbg1, Hbg2, and Hbg3 have undergone marked differentiation in substrate specificity, tissue expression patterns, and enzymatic properties, suggesting that the GH13 α‐glucosidase genes may have experienced distinct functional evolutionary trajectories following gene duplication. However, current understanding of the functional differences among Hbg1, Hbg2, and Hbg3 is derived primarily from biochemical and expression studies in the single species *A. mellifera* . The underlying molecular evolutionary mechanisms, as well as whether these functional differences represent broader evolutionary patterns across Apoidea, remain unclear. In particular, the origin, duplication history, functional divergence, and selection pressures of the α‐glucosidase gene family in *Bombus*, *Osmia*, and other *Apoidea* taxa with different life‐history and feeding characteristics have not yet been systematically investigated.

This study provides the first systematic analysis of the origin, divergence, and selection pressures of the Apoidea α‐glucosidase GH13 gene family from a comparative molecular evolutionary perspective, revealing distinct evolutionary patterns among Hbg1, Hbg2, and Hbg3. The results showed that Hbg1 and Hbg3 experienced different degrees of positive selection, whereas Hbg2 remained relatively conserved. Some positively selected sites overlapped with functionally divergent sites and were concentrated near ligand‐binding regions, suggesting that these sites may be involved in changes in substrate recognition and catalytic properties. These findings indicate that GH13 α‐glucosidases experienced asymmetric selection pressures and functional divergence following gene family diversification, providing new molecular evidence for understanding the adaptive evolution of digestive enzyme gene families in Apoidea.

## 2 Materials and Method

### 2.1 Sequence Collection and Phylogenetic Analyses

The α‐glucosidase genes *Hbg1*, *Hbg2*, and *Hbg3* from the western honey bee, *A. mellifera* , together with annotated GH13 α‐glucosidase genes from *D. melanogaster* and *B. mori* , were used as reference sequences. *Candidate homologs* were searched against the NCBI and FlyBase databases using the TBLASTN algorithm (Altschul et al. 1997). Publicly available genomic or transcriptomic sequences were obtained directly from the corresponding databases, whereas BLAST searches were used to identify and screen candidate GH13 homologs. For species lacking complete target sequences in public databases, sequences were supplemented by PCR amplification or transcriptome assembly. Total RNA was extracted from field‐collected specimens and reverse‐transcribed into cDNA, followed by RT‐PCR amplification using GH13 family‐specific primers and Sanger sequencing. For species with publicly available transcriptomic data, raw reads were downloaded from the NCBI SRA database and assembled using Trinity v2.8.5 (Grabherr et al. 2011). Open reading frames were predicted using TransDecoder v5.5.0 (https://github.com/TransDecoder/TransDecoder), and candidate sequences were subsequently screened by BLAST using *Hbg1*, *Hbg2*, and *Hbg3* as references. Candidate sequences were evaluated based on open reading frame completeness, the presence of conserved GH13 domains, sequence similarity, and phylogenetic clustering. Incomplete, redundant, or ambiguously assigned sequences were excluded before downstream analyses (Table 1). The species sampling in this study was primarily based on phylogenetic coverage and ecological and life‐history diversity, encompassing representative genera from six Apoidea families. We specifically included *Osmia*, *Bombus*, and *Nomia/Megalopta*, which contain lineage‐specific GH13 gene duplication events, to investigate functional divergence following gene duplication. For taxa lacking complete genomic information, sequences were supplemented through SRA transcriptome assembly and RT‐PCR. *Drosophila* and *Bombyx* sequences were used only as homologous references and outgroups.

**TABLE 1:** α‐glucosidase gene sequences information retrieved from the databases.

Family/Order	Name	Species	Accession number	Database
Apidae	AmHbg1	*Apis mellifera*	NM___001040236	NCBI
	AmHbg2	*A. mellifera*	Nm_001040259	NCBI
	AmHbg3	*A. mellifera*	XM_001011608	NCBI
	AfHbg1	*Apis florea*	XM_003692246	NCBI
	AfHbg2	*A. florea*	XM_003691454	NCBI
	AfHbg3	*A. florea*	XM_012484560	NCBI
	BiHbg1	*Bombus impatiens*	XM_003490462	NCBI
	BiHbg21	*B. impatiens*	XM_003493555	NCBI
	BiHbg22	*B. impatiens*	XM_033323439	NCBI
	BiHbg3	*B. impatiens*	XM_033321405	NCBI
	BpHbg1	*Bombus pyrosoma*	XM_043728429	NCBI
	BpHbg21	*B. pyrosoma*	XM_043732547	NCBI
	BpHbg22	*B. pyrosoma*	XM_043731812	NCBI
	BpHbg3	*B. pyrosoma*	XM_043728430	NCBI
Megachilidae	OlHbg11	*Osmia lignaria*	XM_034340438	NCBI
	OlHbg12	*O. lignaria*	XM_034340439	NCBI
	OlHbg2	*O. lignaria*	XM_034325369	NCBI
	ObbHbg11	*Osmia bicornis bicornis*	XM_029195069	NCBI
	ObbHbg12	*O. bicornis bicornis*	XM_029195081	NCBI
	ObbHbg13	*O. bicornis bicornis*	XM_029195094	NCBI
	ObbHbg2	*O. bicornis bicornis*	XM_029187913	NCBI
	MrHbg1	*Megachile rotundata*	XM_003702647	NCBI
	MrHbg2	*M. rotundata*	XM_003703583	NCBI
Halictidae	NmHbg1	*Nomia melanderi*	XM_031980653	NCBI
	NmHbg2	*N. melanderi*	XM_031983218	NCBI
	NmHbg31	*N. melanderi*	XM_031980655	NCBI
	NmHbg32	*N. melanderi*	XM_031980657	NCBI
	MgHbg1	*Megalopta genalis*	XM_033467652	NCBI
	MgHbg2	*M. genalis*	XM_033473320	NCBI
	MgHbg31	*M. genalis*	XM_033467664	NCBI
	MgHbg32	*M. genalis*	XM_033467676	NCBI
Diptera	AdMal	*Anopheles darlingi*	ETN67705	NCBI
	CqMal1	*Culex quinquefasciatus*	XM_001847477	NCBI
	CqMal2	*C. quinquefasciatus*	XM_001851434	NCBI
	DmMalA1	*Drosophila melanogaster*	AAF59089	Flybase
	DmMalA2	*D. melanogaster*	AAF59088	Flybase
	DmMalA3	*D. melanogaster*	AAM50308	Flybase
	DmMalA4	*D. melanogaster*	ABY20547	Flybase
	DmMalA5	*D. melanogaster*	AAF59085	Flybase
	DmMalA6	*D. melanogaster*	AAS64893	Flybase
	DmMalA7	*D. melanogaster*	AAF59084	Flybase
	DmMalA8	*D. melanogaster*	AAF59083	Flybase
	DmMalB1	*D. melanogaster*	AAF53127	Flybase
	DmMalB2	*D. melanogaster*	AAF53128	Flybase
Lepidoptera	BmMal2	*Bombyx mori*	XM_012694492	NCBI
	BmMal3	*B. mori*	NM_001195462	NCBI
	BmMal4	*B. mori*	XM_004930823	NCBI

*Note:* Family, species, gene name, accession number, and database of these sequences were all listed.

We also obtained transcriptomic data for the following bee species from the NCBI SRA database: *Andrena cineraria* (*SRR6148365*), *Andrena camellia* (*SRR8335251*), *Colletes gigas* (*SRR10765021*), *Melitta haemorrhoidalis* (*SRR1503012*), and *Dasypoda hirtipes* (*SRR1503016*) (Table 2). Use fastq‐dump (Cock et al. 2010) from sratools v2.11.0 (Leinonen et al. 2011) to split the downloaded paired‐end (PE) transcriptomic data; subsequently, perform quality control using fastp v0.20.1 (Chen et al. 2018) (default parameters) for quality control, filtering low‐quality reads, and other preprocessing; then perform de novo assembly using Trinity v2.8.5 and extract the longest transcripts. TransDecoder v5.5.0 predicts open reading frames (ORFs) from the Trinity assembly results and translates the predicted ORFs into amino acid sequences. We used Diamond v0.9.24.125 (Buchfink et al. 2015) to perform rapid homology alignment of the predicted protein sequences against the UniProt protein database, identifying transcripts with known protein homology, and then used TransDecoder to determine the most likely coding regions. The assembly data was organized into a database for BLAST searches to obtain homologous sequences for Hbg1, Hbg2, and Hbg3.

**TABLE 2:** Sequences information was obtained in this study.

Family/Order	Species	Name	Collecting Information/transcripts	Access methods
Melittidae	*Macropis* sp.	*MsHbg1*	2021.06.18	PCR + Sanger
		*MsHbg2*	Ankang, China	
		*MsHbg3*		
	*Melitta haemorrhoidalis*	*MhHbg1*	SRR1503012	Transcriptome assembly + BLAST
		*MhHbg2*		
		*MhHbg3*		
	*Dasypoda hirtipes*	*DhHbg1*	SRR1503016	Transcriptome assembly + BLAST
		*DhHbg2*		
		*DhHbg3*		
Colletidae	*Hylaeus* sp.	*HsHbg1*	2021.6.17	PCR + Sanger
		*HsHbg2*	Ankang, China	
		*HsHbg3*		
	*Colletes gigas*	*CgHbg1*	SRR10765021	Transcriptome assembly + BLAST
		*CgHbg2*		
		*CgHbg3*		
Andrenidae	*Andrena cineraria*	*AciHbg1*	SRR6148365	Transcriptome assembly + BLAST
		*AciHbg2*		
		*AciHbg3*		
	*Andrena camellia*	*AcaHbg1*	SRR8335251	Transcriptome assembly + BLAST
		*AcaHbg2*		
		*AcHbg3*		

*Note:* Family, species, gene name, collecting information, and access methods of these sequences were all listed.

The homologous sequences retrieved using the above methods were manually checked using BioEdit v7.2.5 (Hall 1999) to remove incomplete and redundant sequences, and were renamed based on sequence conservation. This resulted in a final set of 63 conserved GH13 family amino acid sequences. Multiple sequence alignment was performed using MAFFT v7.149b (Katoh and Standley 2013) with the FFT‐NS‐2 algorithm, using the following parameters: gap opening penalty of 1.53 and offset value of 0.0. Since the α‐glucosidase family sequences are highly conserved and rich in information‐rich sites, conservative sites were extracted from the alignment results and gap regions were processed using Gblocks v0.91b (Talavera and Castresana 2007; Castresana 2000) for subsequent phylogenetic analysis. The following parameters were used in Gblocks to extract conservative sites and process gap regions: Conservative sites/minimum number of sequences for flanking sites: 27/27, maximum number of consecutive non‐conserved sites: 8, minimum block length: 10, allow gap sites (with half).

We used ModelFinder (Kalyaanamoorthy et al. 2017) to identify the optimal evolutionary model and selected the LG + G model as the best‐fitting model based on the Bayesian Information Criterion (BIC). The software used was RAxML 8.2.12 (Stamatakis 2014) and MrBayes 3.2.7a (Ronquist et al. 2012). Use FigTree 1.4.0 (Tree.bio.ed.ac.uk/software/figtree) to visualize phylogenetic trees. We used the online phylogenetic tree editor ITOL v6 (Letunic and Bork 2024) to modify the font, color, and significant digits of the constructed ML and BI trees, and finally exported PNG files.

### 2.2 Estimation of Evolutionary Divergence Times

In order to clarify the origin and evolution history of the α‐glucosidase family of Apoidea, we used the Reltime (Tamura et al. 2012) method in MEGA7 (Kumar et al. 2016) to analyze the expansion history of the GH13 gene family. Considering that evolutionary rates may differ among lineages within the GH13 gene family, the RelTime method, which does not require the assumption of a strict molecular clock, was used to estimate node divergence times among lineages evolving at different rates. *B. mori* BmMal2, BmMal3, and BmMal4 were used as outgroup sequences, whereas published divergence times for *D. melanogaster* GH13 genes were used as secondary calibration information. Specifically, the divergence times between DmMalA2 and DmMalA3, and between DmMalA4 and DmMalA5, were approximately 155 Ma, whereas that between DmMalB1 and DmMalB2 was approximately 84 Ma (Li et al. 2017). Given that these calibration points were derived from Dipteran insects that are phylogenetically distant from Apoidea, their application to divergence‐time estimation of the Apoidea GH13 gene family may introduce additional uncertainty. Therefore, the absolute divergence times obtained in this study were treated as approximate temporal estimates and were primarily used to compare the relative temporal order of divergence among the Hbg1, Hbg2, and Hbg3 subfamilies and lineage‐specific duplication events, rather than being interpreted as precise absolute divergence ages.

### 2.3 Analysis of Gene Structure and Chromosomal Collinearity of the GH13 α‐Glucosidase Gene Family in the Superfamily Apidae

To investigate whether the GH13 gene family in the Apoidea superfamily underwent structural diversification following gene duplication during evolution, we compared the structural differences and conservation of splicing sites and exon–intron structures among family members, thereby providing evidence for the origin and diversification of the gene family. We also examined the synteny analysis of Hbg within Apoidea. We obtained genomic annotation data for relevant species from Ensembl Metazoa (Yates et al. 2022) and the NCBI Genome Database, and performed colocalization analysis using the Genomicus platform (Louis et al. 2013) to determine its chromosomal distribution and relationships with neighboring genes. For genomes not available on Ensembl (non‐model species such as *Andrenidae*, *Melittidae*, and *Colletidae*), we used the BLAST tool to search for orthologs in the NCBI database and further confirmed their homology through phylogenetic analysis.

### 2.4 Analysis of Functional Divergence in the GH13 Family of α‐Glucosidases in Apoidea Following Gene Duplication

To assess whether functional divergence occurred among different subfamilies of the GH13 α‐glucosidase gene family in the Aopidae following a gene duplication event, was used DIVERGE 3.0 (Gu et al. 2013) to perform a Type I functional divergence analysis. The degree of functional divergence is represented by the functional divergence coefficient *θ*. If *θ* is significantly greater than 0 (LRT test, *p* < 0.05), it indicates that functional divergence occurred between subfamilies during the expansion of the gene family. Additionally, a *z*‐test was used to predict potential amino acid sites driving functional divergence within the gene family. If the *p*‐value was greater than 70%, the site was considered a functional divergence site. Sites with posterior probabilities greater than 0.70 were considered potential functional divergence sites, whereas sites with posterior probabilities greater than 0.90 were regarded as high‐confidence functional divergence sites and marked with an asterisk (*).

### 2.5 Analyses of Selection Pressure of α‐Glucosidase GH13 Family in Apoidea

We analyzed the selection pressure on the GH13 family in Apoidea and calculated the ratio of non‐synonymous (*d*_N_) to synonymous substitutions (*d*_S_) (*ω* = *d*_N_/*d*_S_) by the CodeML program in the PAML 4.8 package (Yang 2007). The models used in the site model were M0 (model = 0, NSsites = 0), M1a (model = 0, NSsites = 1), M2a (model = 0, NSsites = 2), M3 (model = 0, NSsites = 3), M7 (model = 0, NSsites = 7), M8 (model = 0, NSsites = 8). The branch‐specific model uses one‐ratio model (model = 0, NSsites = 0), two‐ratio model (model = 2, NSsites = 0), and free‐ratio model (model = 1, NSsites = 0). Compared with the alternative hypothesis ModelA (model = 2, NSsites = 2, fix_ω = 0, *ω* = 1.5) and the zero‐hypothesis model ModelA null (model = 2, NSsites = 2, fix_ω = 1, *ω* = 1) in the branch‐specific model, the significance test was tested by likelihood ratio test (LRT) and the posterior probability was calculated by BEB test to screen the positive selection sites in the foreground branch.

### 2.6 Three‐Dimensional (3D) Homology Modeling and Ligand‐Binding Prediction

To investigate the relationship between functional divergence and positively selected sites, as well as the three‐dimensional structural characteristics of the α‐glucosidase family proteins in *A. mellifera* , homology modeling was performed for AmHbg1, AmHbg2, and AmHbg3. Protein three‐dimensional structures were predicted and analyzed using the Phyre2 server (http://www.sbg.bio.ic.ac.uk/phyre2) (Kelley et al. 2015), and structural models were selected based on template coverage, sequence identity, and modeling confidence. Following model generation, 3DLigandSite (http://www.sbg.bio.ic.ac.uk/3dligandsite) was used to predict potential catalytic sites and ligand‐binding sites of α‐glucosidases (Wass et al. 2010). The three‐dimensional structure of AmHbg1 was visualized using PyMOL 2.5.2, with the predicted ligand‐binding sites annotated. Furthermore, DIVERGE 3.0 was used to compare functionally divergent sites with positively selected sites, and the sites identified by both analyses were mapped onto the three‐dimensional structure of AmHbg1 to investigate their potential relationships with protein–ligand binding regions and the overall spatial structure of the protein.

## 3 Results

### 3.1 Acquisition and Alignment of Apoidea α‐Glucosidase Gene Sequence

By searching the NCBI database for α‐glucosidase gene sequences from Hymenoptera, we identified candidate members of the GH13 α‐glucosidase family across the sampled species. For species in the families Apidae, Melittidae, and Colletidae for which complete GH13 gene sequences were not found in the database, through PCR amplification and Sanger sequencing, complete GH13 sequences were obtained from *Macropis* sp. and *Hylaeus* sp. In addition, to supplement the data, we downloaded raw transcriptomic sequencing data for five species from the NCBI SRA database: *A. cineraria* (SRR6148365), *A. camellia* (SRR8335251), *C. gigas* (SRR10765021), *M. haemorrhoidalis* (SRR1503012), and *D. hirtipes* (SRR1503016). After assembly and open reading frame prediction, these data were used for gene screening. Transcriptomic data were assembled using Trinity v2.8.5 (default settings). After extracting the longest transcripts, open reading frames (ORFs) were detected using TransDecoder v5.5.0; Diamond v0.9.24.125 was used to align the assembled transcripts with the UniProt protein database to assist in ORF prediction. Subsequently, Hbg1/Hbg2/Hbg3 and *D. melanogaster* (DmMals) were used as auxiliary references for sequence alignment validation, while *B. mori* (BmMal2/BmMal3/BmMal4) served as a distantly related outgroup for phylogenetic analysis. Homologous sequences were identified through BLAST searches against the NCBI database and locally assembled transcriptome databases. By integrating database searches (published genomic sequences of Apidae, Megachilidae, and Halictidae from NCBI), RT‐PCR amplification, and transcriptome assembly data, we identified 63 conserved GH13 family nucleotide sequences from six families within the Superfamily Apoidea. Therefore, these 63 sequences constitute a representative dataset covering six families for reconstructing the evolutionary history of the GH13 gene family, rather than a quantitatively balanced survey of species diversity among families. This sampling design primarily serves the analysis of phylogenetic relationships, gene duplication events, and lineage‐specific functional divergence. The retrieved sequences were manually annotated and renamed based on sequence similarity and phylogenetic placement before subsequent multiple sequence alignment. The results indicate that the α‐glucosidase gene sequences of the GH13 family exhibit high similarity, and the α‐Amy domain associated with the catalytic function of the GH13 family is highly conserved. Therefore, although α‐glucosidases have undergone gene family expansion and subsequent evolutionary divergence within the Apidae family, the proteins they encode retain a highly conserved sequence structure, thereby maintaining the fundamental catalytic function of the GH13 family. This stands in sharp contrast to the loss of catalytic function observed in the mammalian GH13 family members 4F2hc (CD98 heavy chain) and rBAT (b0, + amino acid transporter‐associated protein) (Gabriško and Janeček 2009; Palacín 1994). On the other hand, when comparing Hbg1, Hbg2, and Hbg3, there are varying degrees of sequence length differences in the non‐essential regions of the α‐Amy domain. These differences may lead to variations in the substrates and activity of the α‐glucosidase, thereby enabling unique digestive functions.

### 3.2 Phylogenetic Relationship of α‐Glucosidase GH13 Family in Apoidea

**FIGURE 1:**
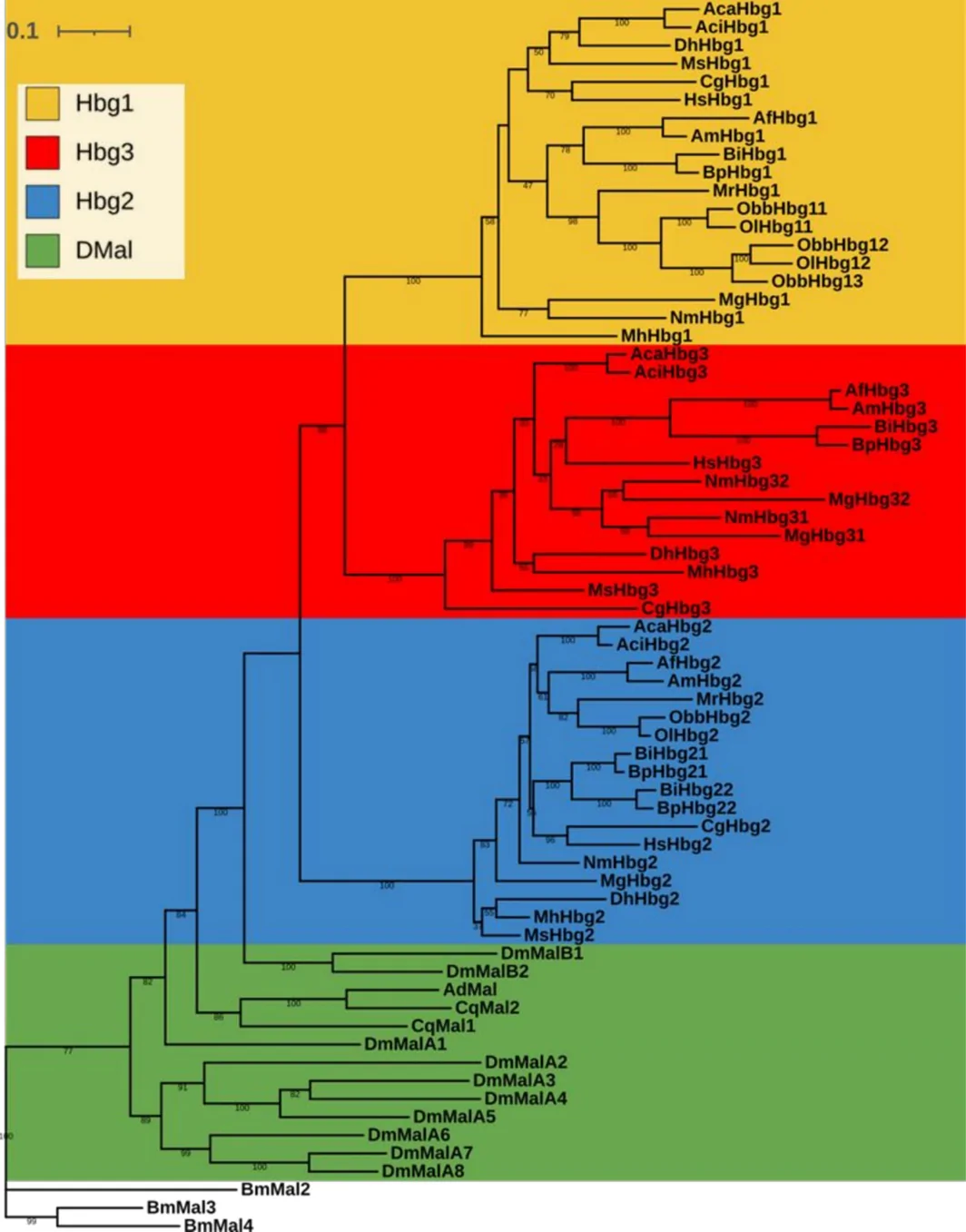
Phylogenetic relationships within the Apoidea α‐glucosidase GH13 family. We used the Maximum Likelihood (ML) method to construct a phylogenetic tree comprising 79 sequences from the superfamily Apidae and other representative insect groups. The scale in the figure represents the amino acid substitution rate, which is the average number of substitutions occurring at each amino acid site. Additionally, DmMal refers to the GH13α‐glucosidase from Diptera.

Based on the selected GH13 α‐glucosidase sequences, a phylogenetic tree was constructed of the GH13 family in Hymenoptera and Diptera using the Bayesian method (BI) (Figure 1). This tree reveals that the GH13 family in Hymenoptera can be divided into three distinct subfamilies: Hbg1, Hbg2, and Hbg3. Hbg1 and Hbg3 cluster together on the phylogenetic tree, while Hbg2 forms a separate branch. The significant genetic distance between Hbg2 and the other two subfamilies suggests that it followed a distinct evolutionary pathway and likely underwent earlier phylogenetic divergence. Furthermore, following the emergence of the ancestral gene for various α‐glucosidases, independent and species‐specific gene duplications occurred in some groups during subsequent evolution, in addition to the gene duplication events that occurred earlier. For example, within the genus Osmia of the Megachilidae family, the Hbg1 gene underwent a lineage‐specific duplication, resulting in the formation of the Hbg11 and Hbg12/13 subfamilies (see the yellow branch in Figure 1). In contrast, only a single copy of the Hbg1 gene has been identified in other species. The Hbg3 gene has duplicated into two copies within the Apidae family, whereas other species possess only a single copy of the Hbg3 gene. These phenomena reflect how the ancestors of different species were influenced by dietary requirements and demonstrate that the adaptive evolution of carbohydrate metabolism enzyme gene families constitutes the molecular basis for the ecological niche differentiation of pollinating insects.

### 3.3 Chromosome Syntenic Analysis of Apoidea α‐Glucosidase Gene

**FIGURE 2:**
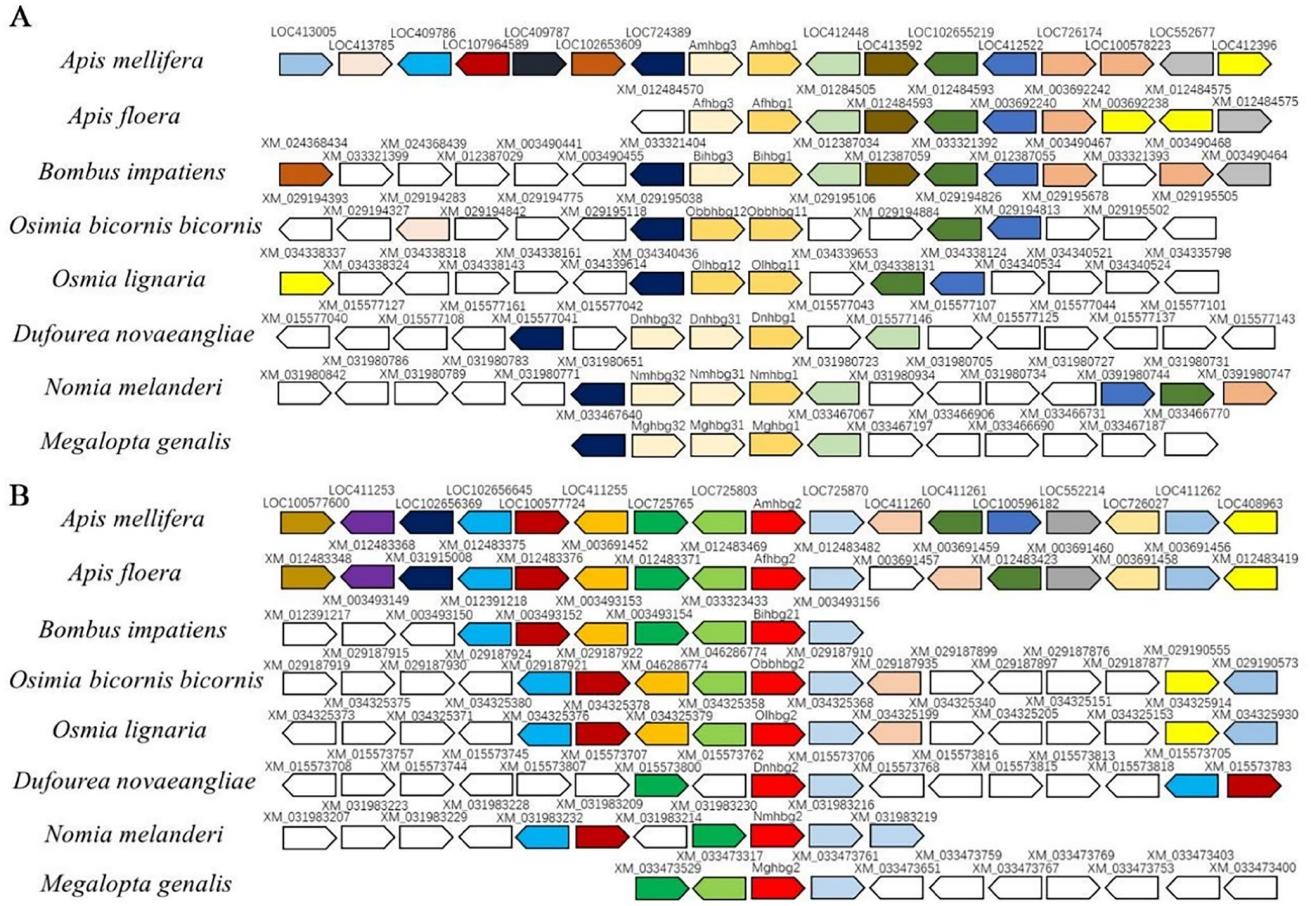
Chromosomal collinear relationship of the GH13 family and its nearby genes in Apoidea. (A) Chromosomal collinearity of *Hbg1*, *Hbg3*, and their neighboring genes in Hymenoptera. *Hbg1* is located in the center and marked in dark yellow, whereas *Hbg3* is marked in light yellow. (B) Chromosomal collinearity of *Hbg2* and its neighboring genes in Hymenoptera. *Hbg2* is located centrally and marked in dark yellow. Homologous genes are indicated by the same color, with different colors representing different homologous gene groups. Regions without homologous genes are marked in white. Gene positions do not represent the actual scale on the chromosomes.

To distinguish the gene duplication types of *Hbg1*, *Hbg2*, and *Hbg3* and infer their mechanisms of origin, we performed a chromosomal colinearity analysis using *AmHbg1*, *AmHbg2*, and *AmHbg3* from the *A. mellifera* genome as reference genes. We found that the GH13 family α‐glucosidases *Hbg1* and *Hbg3* are arranged in tandem on the same chromosome, whereas *Hbg2* is located on a different chromosome (no syntenic), indicating that *Hbg1/Hbg3* and *Hbg2* originated from different gene duplication mechanisms: the former via local tandem duplication, and the latter via interchromosomal duplication or translocation (Figure 2). The genomes of Lepidoptera insects, represented by the silkworm *B. mori* , contain five GH13 family genes distributed across two distinct chromosomal locations; similarly, the GH13 family genes in fruit flies are located on two different chromosomes, suggesting that α‐glucosidase genes arose through the repeated duplication of two sets of genes. These observations suggest that similar patterns of GH13 gene duplication and chromosomal organization may have been conserved across distantly related insect lineages. Furthermore, the distribution of genes near the single‐copy *Hbg1* and *Hbg3* genes is highly similar, and the positions of genes near the single‐copy *Hbg2* gene are also conserved. On the other hand, α‐glucosidase genes in the families Vespidae and Apidae exhibit a bichromosomal copy distribution, and their gene arrangement patterns are similar to those observed near single‐copy α‐glucosidase genes in other species, synteny. The results indicate that α‐glucosidase and its adjacent genes exhibit a significant chromosomal synteny, suggesting that during the evolution of Hymenoptera, the diversification of the GH13 family occurred relatively early, while the subsequent duplication of α‐glucosidase genes exhibited a certain degree of specificity.

### 3.4 Analysis of Gene Structure of α‐Glucosidase GH13 Family in Apoidea

**FIGURE 3:**
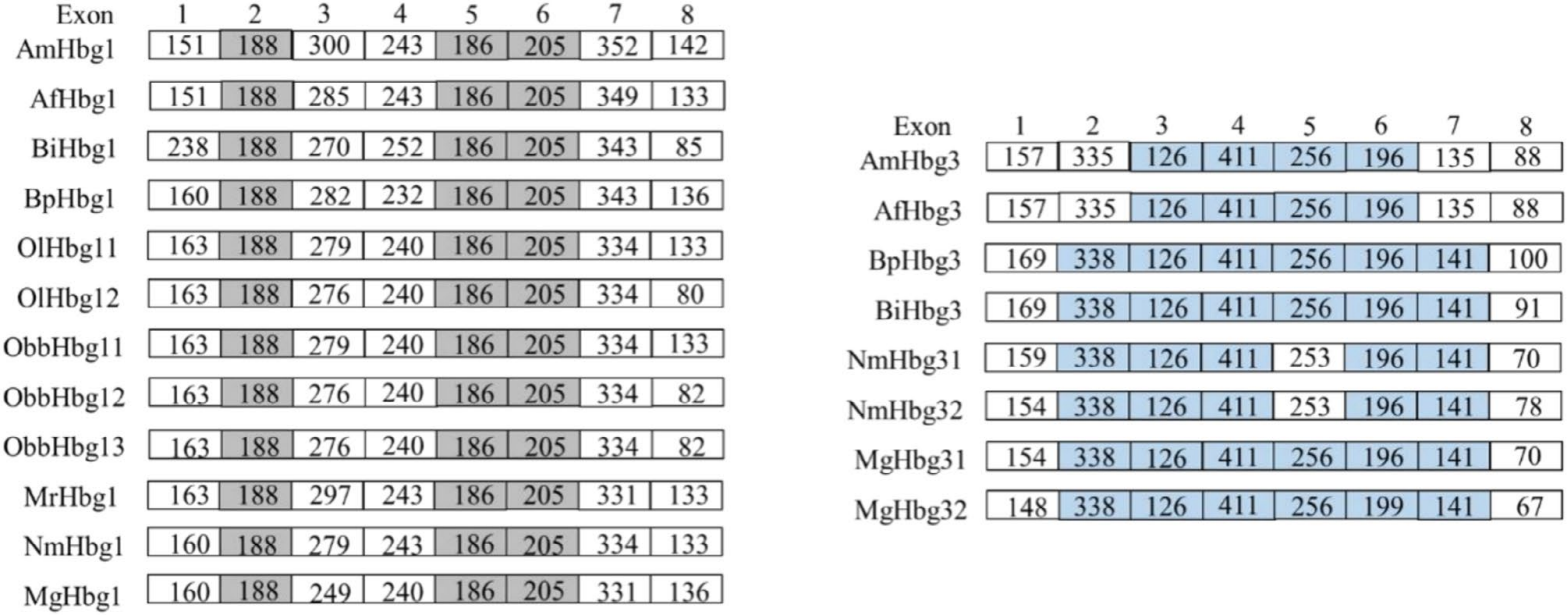
Exon structure of the GH13 family genes in Apoidea. The length of each exon is indicated by the numbers in the boxes; similar exon regions are shown in the same color; the exons are not drawn to scale. Af, *Apis florea* ; Am, *Apis mellifera* ; Bi, *Bombus impatiens* ; Bp, *Bombus pyrosoma* ; Mg, *Megalopta genalis* ; Mr, *Megachile rotundata* ; Nn, *Nomia melanderi* ; Obb, *Osmia bicornis bicornis*; Ol, *Osmia lignaria* .

The structure of the newly generated gene coding region typically changes upon duplication, affecting the amino acid composition of the encoded protein or the intron–exon structure, thereby leading to functional diversification among members of the gene family. To elucidate the structural differences among the Hbg1, Hbg2, and Hbg3 subfamilies, we retrieved the α‐glucosidase gene coding sequences and genomic localization data for representative species from six families of bees from the NCBI database. We analyzed splice‐site conservation and patterns of structural variation, and used Geneious v4.8.5 (Kearse et al. 2012) to compare differences in gene structure. The results showed that all Hbg2 genes in the Hymenoptera GH13 family contain only one exon, whereas Hbg1 and Hbg3 each contain eight exons (Figure 3), suggesting that Hbg2 may have been generated by the reinsertion of ancestral gene mRNA into the genome during the reverse transcription process. The exon composition of the Hbg1 subfamily varies significantly, with only three exons exhibiting conserved lengths. Among these, the splicing patterns of eight exons in OlHbg11 and ObbHbg11 are identical, while those of OlHbg12 and ObbHbg12 are identical except for the 3′‐terminal exon. These paralogous genes have retained their conserved gene structures following duplication. With the exception of exon 3 and the terminal 3′ exon, the remaining six exons of OlHbg12 and ObbHbg12 share the same splicing boundaries and lengths. The exon composition of the Hbg3 subfamily is relatively conserved, with most members exhibiting a splicing pattern consisting of six conserved exons. In particular, the splicing patterns of AmHbg3 and AfHbg3 in *A. mellifera* are identical, whereas the splicing patterns of exons 2 and 7 in Hymenoptera insects differ from those observed in other species within the Apidae family (such as *Bombus*, *Osmia*, and *Nomia*). In addition, the *Hbg3* gene has undergone duplication in the Apidae family, resulting in two paralogous copies (*Hbg31* and *Hbg32*). These two copies exhibit highly conserved gene structures; apart from length variations at the 5′ and 3′ ends, their exon splicing patterns are identical.

### 3.5 The Evolutionary Divergence Time of the GH13 Family of α‐Glucosidases in Apoidea

**FIGURE 4:**
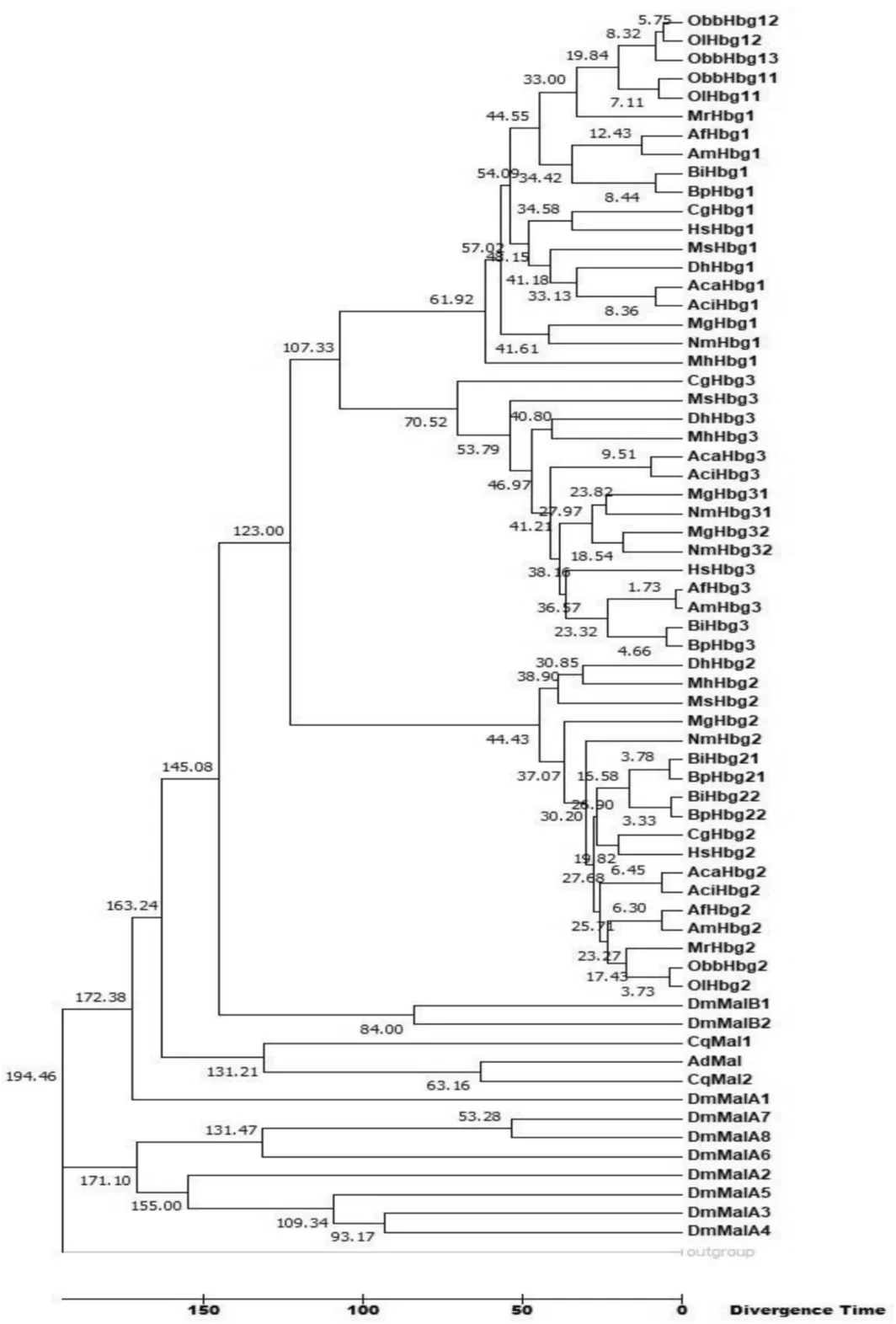
A temporal phylogenetic analysis of the Apoidea GH13α‐glucosidase family was conducted using the RelTime method. Based on phylogenetic relationships, sequence similarity, and chromosomal synteny analysis, genes clustered on the same subfamily branch and showing syntenic conservation across different species were identified as orthologs (Hbg1, Hbg2, Hbg3 branches), while multiple copies of a gene resulting from duplication within the same species were identified as paralogs.

Based on the above studies, the structural characteristics and expression patterns of the α‐glucosidase genes in the GH13 family of Hymenoptera have been largely elucidated. Orthologous and paralogous relationships were inferred by integrating phylogenetic clustering, sequence similarity, and synteny information where genomic locations were available. Genes from different species were regarded as putative orthologs when they clustered within the same Hbg1, Hbg2, or Hbg3 subfamily and showed conserved local synteny. In contrast, multiple copies within the same species that formed species‐specific clades were regarded as putative paralogs derived from lineage‐specific duplication events. Accordingly, the Hbg1, Hbg2, and Hbg3 genes in different sampled bee species were treated as putative orthologous groups, whereas the duplicated copies in *Osmia*, *Bombus*, and *Halictidae* were treated as paralogous copies. The expansion of the GH13 family in Apoidea shows some temporal overlap with the evolutionary history of angiosperms: the origin of the family (145 MYA) and the divergence of *Hbg2* (123 MYA) correspond to the early radiation of angiosperms; the *Hbg1/Hbg3* duplication (107 MYA) coincided with the Late Cretaceous explosion of angiosperm species; whereas the recent duplication events in the genera *Apis* and *Bombus* (17–20 MYA) may have responded to mid‐period climate shifts and the diversification of host plants (Kumar et al. 2016; Tamura et al. 2012) (Figure 4). In the analysis, the divergence times of *D. melanogaster* GH13 genes were used as secondary calibration information. The divergence time between DmMalA2 and DmMalA3/A4/A5 was estimated at 155 MYA, whereas that between DmMalB1 and DmMalB2 was estimated at 84 MYA (Li et al. 2017). Based on the RelTime analysis, the early divergence of the Apoidea GH13 α‐glucosidase family was estimated at approximately 145.08 MYA, followed by the divergence of the Hbg2 family at approximately 123 MYA. The Hbg2 family in *Bombus* subsequently underwent a further divergence approximately 16.58 MYA, giving rise to two subfamilies, Hbg21 and Hbg22. The duplication of the Hbg1 and Hbg3 families occurred approximately 107.33 MYA. Within this lineage, the Hbg1 family in the family Apidae subsequently underwent a duplication event, generating two subfamilies, Hbg11 and Hbg12/13, at approximately 19.84 MYA. The Hbg3 family in the family Anthophoridae subsequently underwent a second duplication event, giving rise to two subfamilies, Hbg31 and Hbg32, at approximately 27.97 MYA.

### 3.6 Functional Diversification of the GH13 Family of α‐Glucosidases in Apoidea

Following the divergence of the *Hbg1*, *Hbg2*, and *Hbg3* subfamilies in Hymenoptera, their gene structures have undergone substantial changes; however, whether these subfamilies exhibit functional divergence remains unclear. To determine whether functional differences exist among the subfamilies of the GH13 family, we performed a Type I functional diversification (FD I) analysis of the GH13 family in Hymenoptera using Diverge 3.0 (Gu et al. 2013). Compared with *DMal* in fruit flies, the functional diversification coefficients for *Hbg1*, *Hbg2*, and *Hbg3* in Hymenoptera were all significantly greater than 0, indicating that the GH13 family in Hymenoptera exhibits diversity. Among these, Hbg1 and Hbg3 had the highest functional diversification coefficients, reflecting that these two subfamilies have undergone significant functional evolution under positive selection since their duplication (Table 3).

**TABLE 3:** Type I functional divergence (FD I) of GH13 alpha‐glucosidase family of Apoidea.

FD	Subfamilies	Coefficient *θ* ± SE	*p*	Functional diverging sites
Type I FD	Hbg1 vs. Hbg3	0.409600 ± 0.029119	*p* < 0.01	43S, 63G, 69L, 72I, 78V*, 87F, 96T, 102D, 108K, 116L*, 118L, 132M*, 134I*, 144K, 174V, 182F, 183H, 185G*, 221D*, 229I, 232V, 242S, 244D, 247P, 249G, 254L*, 259S, 261N, 266K*, 267D*, 268Q, 271T, 274L*, 283D, 284N*, 295V, 296L, 298T*, 302S, 308L*, 322K, 324I, 335Q, 336F, 339I*, 347T*, 357M, 370P, 373A, 376M*, 377I, 378M*, 379L*, 387A*, 393E, 400N, 407D*, 408V, 423N, 429I*, 442H*, 446K, 447S*, 468L, 472R, 474R, 477L, 480G, 490T*, 492L, 497Q*, 498S*, 504S, 505L, 506L*, 507I, 509F, 513N, 517D*, 527N*, 531Y*, 553P, 557S, 560V
	Hbg1 vs. Hbg2	0.277600 ± 0.026314	*P* < 0.01	46I, 53K, 59F, 78V*, 88K*, 101E, 104T*, 125D, 128K, 132M*144K, 146K*, 172L, 174V*, 179G, 182F, 189F, 191F, 218F, 220L*, 221D, 222K*, 224I, 239A*, 249G*, 256L*, 261N*, 266K, 274L, 275V, 282V*, 285Y*, 289N, 333P, 365V, 370P*, 381M*, 386V, 387A, 408V, 477L*, 527N*
	Hbg1 vs. DMal	0.292800 ± 0.032471	*P* < 0.01	43S, 60I, 65T, 126Q*, 132M, 187K, 191F, 204R, 210E, 211E*, 221D, 225D*, 229I, 253N, 262H, 266K, 272Y*, 285Y*, 298T*, 307T, 357M*, 370P, 376M, 381M, 386V*, 395I, 407D*, 505L*, 510S, 552I, 553P*
	Hbg3 vs. Hbg2	0.325600 ± 0.029512	*P* < 0.01	46I, 53K*, 63G*, 72I, 87F, 89D, 101E, 104T, 110Q, 125D*, 134I*, 162P, 176N*, 185G*, 189F, 204R, 215I, 220L*, 242S*, 254L*, 256L, 257H, 269P, 270E, 282V*, 298T, 308L*, 347T, 363V, 372R, 376M*, 377I*, 382I, 407D*, 429I*, 442H, 47 2R, 490T*, 498S, 501E*, 504S*, 555D, 557S
	Hbg3 vs. DMal	0.208000 ± 0.035987	*P* < 0.01	60I, 63G, 116L, 131Q, 185G, 204R, 225D, 270E, 272Y*, 335Q, 442H, 468L, 501E, 504S, 527N, 560V
	Hbg2 vs. DMal	0.272000 ± 0.038875	*P* < 0.01	50K, 59F*, 65T, 112L, 126Q, 187K, 206S*, 211E*, 220L, 222K, 225D, 269P, 272Y*, 281F, 298T, 337K, 349Q, 350N, 376M, 377I*, 395I, 407D, 408V, 444S, 553P

*Note:* Functional divergences for pairwise comparisons are shown as value ± standard error. Critical amino acid sites detected relating to FD with *p* > 70%, and *p* > 0.9 were indicated with *.

Subsequent studies revealed that most of the potential functional divergence sites are located within the conserved catalytic domain of the GH13 family, suggesting that these sites may influence the catalytic activity and enzymatic properties of α‐glucosidases in the Apidae family. This analysis also provides a theoretical foundation for future research, aiding in the further identification, at the physiological level, of the key amino acid sites responsible for the substrate‐specific differentiation of α‐glucosidases in the subfamily *Vespinae*.

### 3.7 Analysis of Selection Pressure of α‐Glucosidase GH13 Family in Apoidea

To investigate the molecular evolutionary mechanisms underlying functional divergence of α‐glucosidases in the Apoidea superfamily, we estimated the ratio of non‐synonymous to synonymous nucleotide substitution rates (*d*_N_/*d*_S_ or *ω*). Using the CodeML module in PAML 4.8 (Yang 2007), we performed likelihood ratio tests (LRTs) with site‐specific and branch‐specific models. Under the most basic M0 model (which assumes that *ω* remains constant across sites and branches), the *ω* value for the entire GH13 family of α‐glucosidases in Apoidea was 0.23878, indicating that significant purifying selection was present at most sites during the evolution of α‐glucosidases (Table 4). A comparison of the M0 model with the M3, M1a, and M2a models revealed that the GH13 family of α‐glucosidases in the Apoidea superfamily is subject to varying degrees of selective pressure at different sites. The M7 model assumes that *ω* follows a beta distribution constrained between 0 and 1, whereas the M8 model adds an additional category with *ω* > 1. The results indicated that the M8 model significantly outperformed the M7 model, with a total of 15 potential positive selection sites identified and an *ω* value of 1.38623. These sites were primarily distributed within the catalytic domain of the GH13 family genes. Diverge analysis revealed that the 215I, 349Q, 372R, 373A, 386V, and 527N sites are associated with functional divergence in Hymenoptera α‐glucosidases. In summary, during the evolution of the Hymenoptera GH13 family, the primary selective pressure was purifying selection. However, there were significant differences in the selective pressures acting on specific sites within the Hymenoptera GH13 family, and a small number of sites underwent the mutation process described by Darwinian positive selection theory, a process that facilitated the functional diversification of Hymenoptera α‐glucosidase.

**TABLE 4:** Test of positive selection on apoidean GH13 family α‐glucosidase by site model.

Model	Parameters	Comparison	LRT *p*‐value	Positively sites
M0	*ω* = 0.23878 *ω*0 = 0.02334	M0 vs. M3	< 0.01	None
M3	*p*1 = 0.42618 *ω*1 = 0.21371 *p*2 = 0.24857 *ω*2 = 0.60130			
M1a	*p*0 = 0.66242, *ω*0 = 0.14264; *p*1 = 0.33117, *ω*1 = 1.00000	M1a vs. M2a	< 0.01	None
M2a	*p*0 = 0.66242 *ω*0 = 0.14359, *p*1 = 0.32479, *ω*1 = 1.00000, *p*2 = 0.01279, *ω*2 = 2.30898			
M7	*p* = 0.51834, *q* = 1.44109	M7 vs. M8	< 0.01	215I**, 217K, 290K, 310Y, 312E, 372R, 373A*, 385G**, 386V**, 391Y, 349Q*, 527N*, 540T **, 541V**, 544T*
M8	*p*0 = 0.98054, *p*1 = 0.01946, *ω* = 1.38623; *p* = 0.56974, *q* = 1.83880			

*Note:* *ω* means *d*_N_/*d*_S_, * means posterior probability Pb > 95%, ** means Pb > 99%.

To assess the patterns of selective pressure across lineages within the Hymenoptera GH13 family, we performed lineage‐specific analyses using the Codeml program in the PAML 4.8 software package: We treated the single‐rate model (M0) as the null hypothesis (all lineages have the same *ω* value) and the free‐rate model as the alternative hypothesis (*ω* is estimated independently for each lineage), and assessed the heterogeneity of selective pressure using the likelihood ratio test (LRT) (Yang and Nielsen 2002). Results from the single‐ratio model indicate that each branch of the GH13 family within the Apoidea is primarily subject to purifying selection, with an *ω* value of 0.23878. However, results from the free‐ratio model suggest that different branches of the GH13 family within the superfamily Apidae are under varying selective pressures. Free‐ratio models revealed substantial differences in selection pressures among different lineages of the Apoidea GH13 family. The Hbg1 lineage exhibited a markedly elevated *ω* value (*ω* = 33.7879), with a total of 20 positively selected sites detected, indicating a signal of positive selection in this lineage. In contrast, Hbg2 (*ω* = 0.026), Hbg3 (*ω* = 0.0025), and the ancestral lineages of Hbg1 and Hbg3 (*ω* = 0.0024) all exhibited signatures of purifying selection. Furthermore, the Hbg12 subclade, formed by the specific duplication of Hbg1 within the genus Osmia, is subject to positive selection (*ω* = 2.13336), with one copy in each species still under purifying selection, while the remaining copies are subject to positive selection. The free‐ratio model also detected positive selection on Hbg2 and certain branches of this family within the Vespidae (Table 5). In summary, positive selection has played a role in the speciation of the GH13 family during the evolution of Hymenoptera, further influencing the formation of subfamilies resulting from specific duplication, and promoting the evolution of the Hymenoptera α‐glucosidase gene family by altering the substitution patterns at codon sites.

**TABLE 5:** Positive selection on Apoidea GH13 α‐glucosidase family by branch‐specific model.

Model	Parameters	Ln L	LRT *p*‐value
One‐radio model	*ω* = 0.23878	−57,166.635066	
	*ω*Hbg1 = 33.7879		
	*ω*Hbg2 = 0.0264844		
	*ω*Hbg3 = 0.00245742		
	*ω*Hbg1‐Hbg3 = 0.00236336		
Free‐radio model	*ω*OsmiaHbg12‐Hbg13 = 1.37318 *ω*OsmiaHbg12 = 2.13336 *ω*ObbHbg11 = 1.09152	−56,775.746142	< 0.01
	*ω*BpHbg1 = 1.52046		
	*ω*AfHbg1 = 1.81232		
	*ω*AndrenidaeHbg2 = 338.808		
	*ω*OlHbg2 = 999		

*Note:* *ω* means *d*_N_/*d*_S_, *p* < 0.05 represents significant, and *p* < 0.01 represents extremely significant, which is obtained by the LRT test.

The site‐specific model assumes that all lineages in the phylogenetic tree have a consistent evolution rate to detect universal and extensive positive selection, while the branch‐specific model is more accurate and stable and is used to detect positive selection in a branch breakpoint. The branch‐specific model was used to detect the positive selected sites for specific branches of the GH13 family in Apoidea (Table 6). We detected 20 and 31 sites subjected to positive selection from the Hbg1 and Hbg3 lineages, respectively. Eleven sites in the differentiation of Hbg1 and Hbg3 were subjected to positive selection, while Hbg2 was not subjected to positive selection, indicating that Hbg2 is a more conservative class of α‐glucosidase. Among them, 532 T was subjected to positive selection both in the lineages of Hbg1 and Hbg3, 291R and 412C were subjected to positive selection in Hbg1, Hbg1 and ancestral lineages of Hbg3, 286A was subjected to positive selection in Hbg3, Hbg1 and ancestral lineages of Hbg3, and the other positive selection sites were branch‐specific. In addition, 5, 3, and 10 positive selection sites were also subjected in Hbg1, Hbg3, and the differentiation of the Apoidea GH13 family.

**TABLE 6:** Positive selection on Apoidea GH13 α‐glucosidase family by branch‐site model.

Model	Foreground	*p*	Positively selected sites
Model vs. Model A Null	Hbg1	< 0.01	9L*, 144K*, 242S*, 248L, 259S**, 264L, 291R, 306N, 327A, 362R, 411G*, 412C, 427S*, 453Q, 472R, 475D, 528A, 532T, 560V, 578L
Model vs. Model A Null	Hbg2	> 0.05	259S
Model vs. Model A Null	Hbg3	< 0.01	27N*, 29I*, 123T, 152V, 183H*, 188Q*, 209R, 228R, 249G*, 260L, 262H, 286A, 315S, 322K, 323F, 352V, 374D, 375H*, 381M, 387A, 400N, 423N, 435E, 449L, 503V, 505L*, 506L, 510S*, 514I, 532T, 547P
Model vs. Model A Null	Hbg1 + Hbg3	< 0.01	8V, 25W, 77M, 108K, 181T, 222K*, 233P, 286A, 291R, 412C, 484T
Model vs. Model A Null	Polyphagous Hbg1	< 0.05	252L, 287E, 303S, 335Q, 514T
Model vs. Model A Null	Polyphagous Hbg2	> 0.05	274L
Model vs. Model A Null	Polyphagous Hbg3	< 0.01	24W, 66A, 257H, 293E, 295V, 327A, 398V, 480G, 510S

Apoidea are gregarious, omnivorous pollinators; their seasonal shift between various nectar‐producing plants contrasts sharply with the specialized feeding habits of other groups within the superfamily Apoidea. To explore whether the α‐glucosidase of polyphagous social bees is subjected to positive selection, the branch representing polyphagous social bees was designated as the foreground branch, and a branch‐site model was applied to test for lineage‐specific positive selection. The results showed that the partial sites of Hbg1 and Hbg3 of polyphagous bees are subjected to positive selection. We found five and nine positive selections respectively, and these sites were located in the catalytic domain of the GH13 family, of which 257H, 295V, 480G, and 510S were not only subjected to positive selection but also potential functional differentiation sites of the GH13 family (Table 6). The results demonstrated that some sites of α‐glucosidase in Apoidea were subjected to positive selection because of the changes in living habits, changing the function and catalytic activity of α‐glucosidase to maintain energy metabolism more efficiently and adapt to more complex social habits and food sources. In conclusion, different lineages of the GH13 family in Apoidea are subjected to various selective pressures in the evolution, and the positive selection sites of each branch were also different. Partial sites of Hbg1 and Hbg3 were subjected to positive selection, while Hbg2 was not subjected to positive selection. At the same time, partial sites of Hbg1 and Hbg3 in polyphagous social bees were also subjected to positive selection in different degrees. The differences between these sites impacted the expansion pattern in the evolution of Apoidea's GH13 family, resulting in functional differentiation of the GH13 family of Apoidea.

### 3.8 Homologous 3D Structure Modeling and Spatial Localization of Key Sites

Although the α‐glucosidases Hbg1, Hbg2, and Hbg3 in the family Apidae exhibit high sequence similarity, they show marked differences in substrate specificity and catalytic activity. To investigate the relationship between sequence characteristics and structural divergence among the three proteins, homology modeling was performed to construct three‐dimensional structural models of *AmHbg1*, *AmHbg2*, and *AmHbg3*. The GH13 sucrose hydrolase *BmSUH* from the silkworm ( *B. mori* ) (PDB ID: 6LGH) was identified as the best‐matching structural template for all three proteins. The template coverage of *AmHbg1*, *AmHbg2*, and *AmHbg3* was 91%, 95%, and 96%, respectively, with a Phyre2 modeling confidence of 100% for all three models and sequence identities of 40%, 42%, and 44%, respectively. The high template coverage and modeling confidence indicate that the three proteins have relatively high structural homology with the template (Miyazaki and Park 2020). In addition, all three proteins retained the conserved structural domain of the GH13 family, indicating a high degree of conservation in their overall structural framework. Given that these structures were homology‐modeling predictions rather than experimentally determined structures, they were primarily used for qualitative comparisons of overall structural features and the spatial distribution of relevant sites.

**FIGURE 5:**
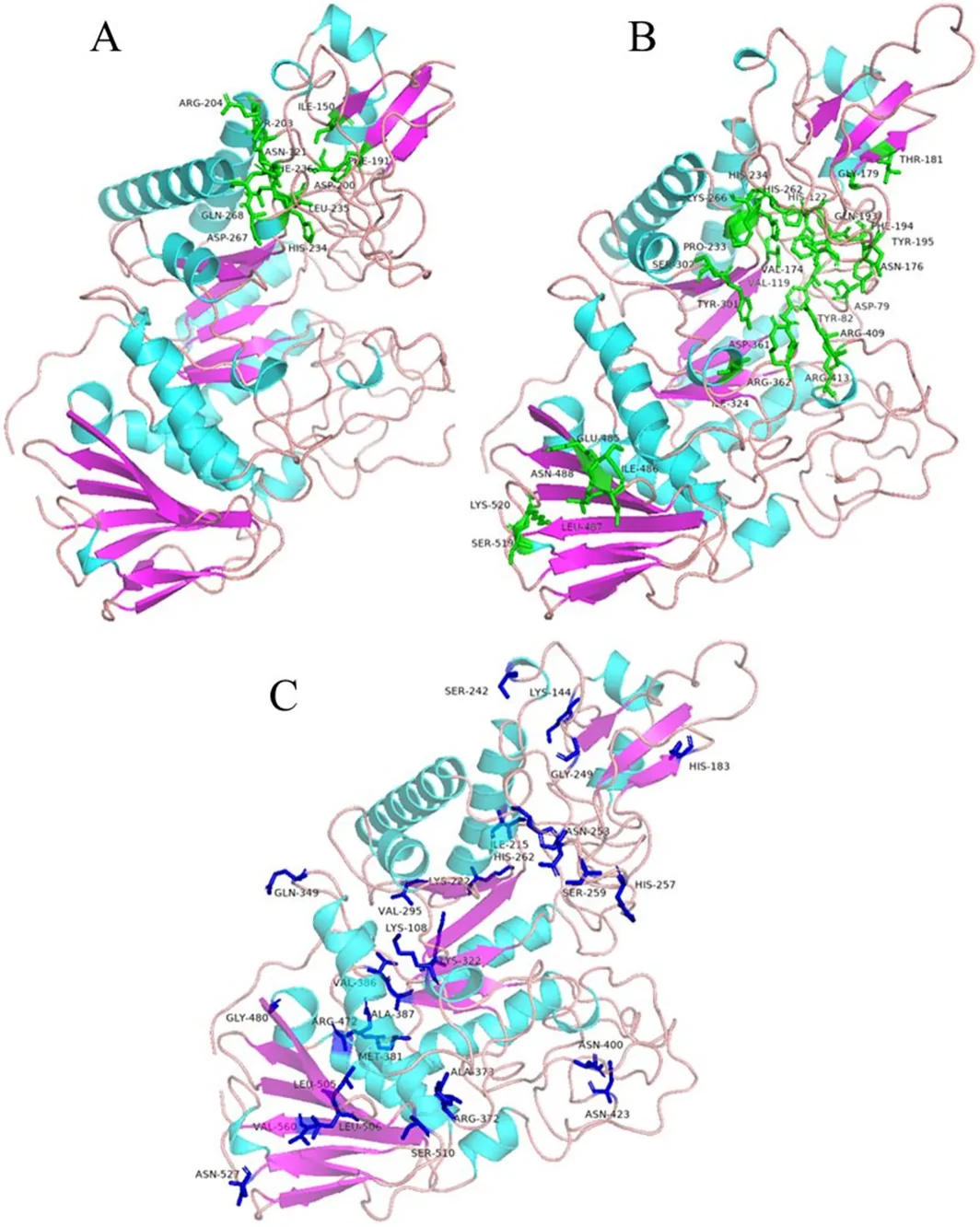
Three‐dimensional structure of α‐glucosidase, showing positive selection sites and functionally divergent residues. (A) The magnesium‐binding site of α‐glucosidase is highlighted in green. (B) The sugar‐binding site of α‐glucosidase is highlighted in green. (C) Sites subject to both positive selection and functional diversification are highlighted in blue. The structural model was obtained via homology modeling using Phyre2 Version 2 (http://www.sbg.bio.ic.ac.uk/phyre2) with the Bt‐sucrose hydrolase BmSUH (PDB ID: 6LGH) from the silkworm as a template; the ligand‐binding site was predicted by 3DLigandSite Version 1.0 (McGreig et al. 2022), and visualization and site labeling were performed using PyMOL Version 2.5.2 (Seeliger and de Groot 2010). Hbg1, Hbg2, and Hbg3 share the same best‐fit model, and the protein structures of the three are highly conserved.

Ligand‐binding sites play a crucial role in the interaction between proteins and their ligands. To identify key sites in the higher‐order structure of Apis α‐glucosidase, this study utilized the 3DligandSite online server (sbg.bio.ic.ac.uk/~3dligandsite) to predict potential binding sites in Apis α‐glucosidase (represented by AmHbg1). We identified one magnesium‐binding domain and two sugar‐binding domains in AmHbg1. We characterized 11 binding sites in the magnesium‐binding domain, 22 binding sites in sugar‐binding domain 1, and 6 binding sites in sugar‐binding domain 2, which correspond to the metal ion coordination required for substrate recognition and catalytic activity (Figure 5A,B). These sites determined the binding of α‐glucosidase to magnesium ions and substrate. Furthermore, 28 sites in Apoidea are not only sites of positive selection in the GH13 family but also potential sites of functional diversification. These 28 sites are mapped onto the α‐glucosidase structure, and they are primarily located near the sugar‐ and magnesium‐binding regions of the α‐glucosidase (Figure 5C). In summary, although only a small number of ligand‐binding sites are targets of positive selection and functional diversification, the spatial distribution of these 28 functionally diversified and positively selected sites indicates that positive selection influences the binding of Apoidea α‐glucosidases to ligands and the spatial conformation of the proteins.

## 4 Discussion

Over the course of their long evolutionary history, Hymenoptera insects have, through adaptive evolution, gradually developed behavioral and morphological traits that facilitate their survival, feeding, and digestion. The primary energy source for insects in the superfamily Apoidea comes from sugars found in nectar and pollen collected from flowering plants. These nectar‐producing plants include members of the *Fabaceae*, *Lamiaceae*, *Rosaceae*, and *Asteraceae* families. As an important carbohydrate hydrolase, α‐glucosidase plays a key role in regulating the digestive and absorptive processes of Apoidea insects. Throughout insect evolution, α‐glucosidases have undergone complex functional diversification across different taxonomic groups, accompanied by gene family expansion. This indicates an adaptive evolutionary relationship between insect α‐glucosidases and sugars and other chemical compounds in food, while also reflecting the adaptive evolution of the digestive system itself.

In the present study, we obtained the evolutionary model of Apoidea α‐glucosidase by analyzing the phylogeny, gene structure, chromosome synteny, functional differentiation and selection pressure of the α‐glucosidase gene family. The ancestral genes of the GH13 family in Apoidea were produced in the early Cretaceous (about 144 MYA), and the pollen of angiosperms was produced in the early stage (about 139–131 MYA) (Doyle 2012). Subsequently, during the radiation and expansion of angiosperms from the late Early Cretaceous to the Late Cretaceous, the ancestral gene of the Apoidea GH13 family underwent two rounds of diversification: *Hbg2* arose around 123 MYA, and *Hbg1* and *Hbg3* were duplicated around 107 MYA. Lineage‐specific duplications subsequently generated *Osmia Hbg11* and *Hbg12* in *Megachilidae*, *Bombus Hbg21* and *Hbg22* in *Apidae*, and *Hbg31* and *Hbg32* in *Halictidae*. Genes from different sampled bee species that clustered within the same *Hbg1*, *Hbg2*, or *Hbg3* clade were regarded as putative orthologs. In contrast, multiple copies forming lineage‐specific clades in *Osmia*, *Bombus*, and Halictidae were regarded as putative paralogs generated by lineage‐specific duplication events. These paralogous copies may have provided a basis for subsequent sequence and functional divergence. In addition, the GH13 family also produced gene loss events, and the Megachilidae lost *Hbg3* during the expansion of the α‐glucosidase gene family (Figure 1). Hbg1 and Hbg3 were tandemly arrayed in the Apoidea GH13 family, but Hbg2 was located at different positions on another chromosome (Figure 2). During millions of years of evolution, the GH13 family in Apoidea had undergone five gene expansions and produced six members of the α‐glucosidase gene family (Figure 4).

Distinct functional divergence and differential selection pressures were observed among the subfamilies of the Apoidea GH13 family, with signals of positive selection detected in Hbg1, Hbg3, and several lineages. The 28 sites identified as both positively selected and potentially functionally divergent were mainly distributed near ligand‐binding regions, suggesting that they may be involved in substrate recognition and changes in catalytic properties. Apoidea taxa exhibit marked differences in sociality, feeding ecology, and patterns of floral resource utilization. Social and relatively broadly feeding bees can exploit more diverse and dynamic pollen and nectar resources, whereas some solitary bees exhibit relatively specialized feeding strategies. Differences in the carbohydrate composition and content of pollen and nectar may also generate distinct nutritional selection regimes (Wcislo and Cane 1996; Wright et al. 2018; Liu et al. 2024). Consistent with this, several positively selected sites were detected in Hbg1 and Hbg3 of omnivorous social bees, and different lineages exhibited distinct selection pressures and patterns of gene duplication, suggesting that food resource utilization strategies may be associated, to some extent, with lineage‐specific diversification of the GH13 family. Furthermore, homology modeling showed that Hbg1, Hbg2, and Hbg3 possess similar three‐dimensional structural models, indicating that the overall structural framework of the Apoidea GH13 family is relatively conserved. Sites associated with positive selection and functional divergence exhibited a discernible spatial distribution pattern and were mainly located near ligand‐binding regions (Figure 5), suggesting that these sites may contribute to the divergence of substrate binding and catalytic properties of GH13 α‐glucosidases. However, given that high *ω* values may be influenced by the limited number of substitutions and sequence alignment uncertainty, these sites should still be regarded as candidate functional residues, and their relationships with ecological adaptation and enzymatic function require further validation. Although the BmSUH model has relatively broad sequence coverage, its sequence identity with the Hbg proteins is approximately 40%. Therefore, the predicted ligand‐binding regions should be interpreted with caution. Measurements of binding affinities and ligand interactions under different substrate conditions are still required to further determine the extent of divergence in ligand‐binding sites among the sampled bee lineages of the GH13 α‐glucosidase family. The study indicates that natural selection over long‐term evolution has driven the emergence and functional diversification of Apoidea α‐glucosidases, thereby facilitating the efficient absorption and metabolism of carbohydrates by Apoidea insects. The evolution of α‐glucosidases reflects the ability of Apoidea insects to adapt to their environment and diet by altering physiological traits.

## 5 Conclusions

By analyzing the functional differentiation and adaptive evolution of α‐glucosidases with digestive functions in honeybees, this study demonstrates that honeybees exhibit specific adaptive differentiation in response to the diversity of their living environments and the critical requirements of their own growth and development. These findings provide molecular evidence that may be associated with the diversification of digestive functions and dietary adaptation in Apoidea.

The results indicate that the α‐glucosidase gene family underwent two rounds of diversification during the early evolutionary history of the Apoidea, giving rise to the GH13 family members Hbg1, Hbg2, and Hbg3, which exhibit distinct expression patterns and functional specializations. The existing GH13 family of α‐glucosidases in the Apoidea are located in different regions of two chromosomes, have undergone at least five gene duplication events, and contain a large number of specific amino acid sites that contribute to functional diversification. Members of the Bee family of α‐glucosidases have been subject to varying selective pressures during evolution. Hbg1, Hbg3, and certain subclades have been significantly influenced by positive selection, while different sites have been subject to varying selective pressures. Mutations occurring at certain sites under positive selection may have contributed to the functional diversification of the GH13 family. There are 28 amino acid sites, which are not only affected by positive selection but also lead to the functional differentiation of honeybee α‐glucosidase, which are obviously concentrated near the protein‐ligand binding region, affecting the substrate specificity of honeybee α‐glucosidase.

Catalytic activity and three‐dimensional conformation of protein. Further differentiation occurred in the enzymatic activity of some lineage‐specific duplicated genes, which led to the further expansion and functional differentiation of the GH13 family of honeybee family.

## Author Contributions

**Jiaqi Li:** conceptualization (supporting), data curation (equal), methodology (equal), visualization (equal), writing – original draft (lead), writing – review and editing (equal). **Wenmi Wang:** conceptualization (supporting), data curation (equal), methodology (equal), visualization (equal). **Yuanyuan Du:** conceptualization (supporting), visualization (equal). **Junxia Jia:** conceptualization (supporting), methodology (equal), visualization (equal). **Zhaoming Wei:** conceptualization (lead), funding acquisition (lead), methodology (equal), writing – review and editing (equal).

## Funding

This study was supported by the Natural Science Foundation of Shaanxi Province (2015SF251) and College Student Innovation and Entrepreneurship project (S202310718169).

## Conflicts of Interest

The authors declare no conflicts of interest.

## Data Availability

Nucleic acid sequences and protein sequences obtained in this study are accessible on Figshare: https://figshare.com/s/3717c0a31b2eb7d13af8.
